# A major quantitative trait locus conferring adult plant partial resistance to crown rust in oat

**DOI:** 10.1186/s12870-014-0250-2

**Published:** 2014-09-27

**Authors:** Yang Lin, Belaghihalli N Gnanesh, James Chong, Gang Chen, Aaron D Beattie, Jennifer W Mitchell Fetch, H Randy Kutcher, Peter E Eckstein, Jim G Menzies, Eric W Jackson, Curt A McCartney

**Affiliations:** Crop Development Centre/Department of Plant Sciences, University of Saskatchewan, 51 Campus Drive, Saskatoon, SK S7N 5A8 Canada; Agriculture and Agri-Food Canada, Cereal Research Centre, 101 Route 100, Morden, MB R6M 1Y5 Canada; General Mills Agriculture Research, 150 N. Research Campus Dr, Kannapolis, NC 28081 USA

**Keywords:** Crown rust, *Puccinia coronata*, Oat, *Avena sativa*, Adult plant resistance, Partial resistance, SNP, QTL

## Abstract

**Background:**

Crown rust, caused by *Puccinia coronata* f. sp. *avenae*, is the most important disease of oat worldwide. Adult plant resistance (APR), based upon partial resistance, has proven to be a durable rust management strategy in other cereal rust pathosystems. The crown rust APR in the oat line MN841801 has been effective for more than 30 years. The genetic basis of this APR was studied under field conditions in three recombinant inbred line (RIL) populations: 1) AC Assiniboia/MN841801, 2) AC Medallion/MN841801, and 3) Makuru/MN841801. The populations were evaluated for crown rust resistance with the crown rust isolate CR251 (race BRBB) in multiple environments. The 6 K oat and 90 K wheat Illumina Infinium single nucleotide polymorphism (SNP) arrays were used for genotyping the AC Assiniboia/MN841801 population. KASP assays were designed for selected SNPs and genotyped on the other two populations.

**Results:**

This study reports a high density genetic linkage map constructed with oat and wheat SNP markers in the AC Assiniboia/MN841801 RIL population. Most wheat SNPs were monomorphic in the oat population. However the polymorphic wheat SNPs could be scored accurately and integrated well into the linkage map. A major quantitative trait locus (QTL) on oat chromosome 14D, designated *QPc.crc-14D*, explained up to 76% of the APR phenotypic variance. This QTL is flanked by two SNP markers, GMI_GBS_90753 and GMI_ES14_c1439_83. *QPc.crc-14D* was validated in the populations AC Medallion/MN841801 and Makuru/MN841801.

**Conclusions:**

We report the first APR QTL in oat with a large and consistent effect. *QPc.crc-14D* was statistically significant in all environments tested in each of the three oat populations. *QPc.crc-14D* is a suitable candidate for use in marker-assisted breeding and also an excellent target for map-based cloning. This is also the first study to use the 90 K wheat Infinium SNP array on oat for marker development and comparative mapping. The Infinium SNP array is a useful tool for saturating oat maps with markers. Synteny with wheat suggests that *QPc.crc-14D* is orthologous with the stripe rust APR gene *Yr16* in wheat.

**Electronic supplementary material:**

The online version of this article (doi:10.1186/s12870-014-0250-2) contains supplementary material, which is available to authorized users.

## Background

Crown rust caused by *Puccinia coronata* Corda f. sp. *avenae* Eriks. is the most economically important disease of cultivated oat (*Avena sativa* L.) [1,2]. Resistant oat varieties are an important control strategy for managing this disease. Loss of an estimated $400 million was prevented from 1995 to 2005 because of the cultivation of resistant oat varieties in Canada [3]. Methods of control have usually been based on single major genes that confer complete resistance based upon a gene-for-gene interaction. These major genes are typically expressed at all plant growth stages and are called seedling resistance genes. It should be noted that adult plant genes (*Lr12*, *Lr13*) have been discovered in the wheat-*Puccinia triticina* pathosystem that are based upon gene-for-gene interaction [4]. The widespread deployment of seedling genes conferring complete resistance has universally resulted in the emergence of new virulent *P. coronata* races [3]. Adult plant partial resistance does not completely prevent sporulation of the fungus, but reduces pustule size, spore production, and extends the latent period [5]. Partial resistance is believed to be more effective in controlling the disease because it promotes coexistence of host and pathogen and thus slows the evolution of pathogen virulence. However, partial resistance is more difficult to use in plant breeding because the breeder must select for quantitative differences in disease reaction. In addition, adult plant partial resistance can also be confused with complete resistance that is only effective against a portion of the pathogen population present in a field nursery.

Major seedling resistance genes are widely used in North American rust resistant oat varieties [6]. Such race-specific resistance genes are relatively easy to utilize in breeding lines but extensive use of seedling resistance genes in breeding programs results in the eventual selection of *P. coronata* races with virulence to those genes. For instance, *P. coronata* races in western Canada defeated previously effective seedling resistance genes such as *Pc38* and *Pc39* in the late 1980s, *Pc48* in 2001 and *Pc68* in 2005 [3]. Carson [7] reported oat cultivars with major gene resistance to crown rust in the U.S.A. generally succumbed to the pathogen in 5 years or less after release. Continued widespread use of cultivars carrying single race-specific seedling genes will likely continue this trend. Research on adult plant resistance (APR) to wheat leaf rust resulted in the identification of the adult plant partial resistance genes *Lr34*, *Lr46*, and *Lr67* [8-10]. *Lr34* has been widely incorporated into many wheat cultivars around the world and since 1966 has not yet been overcome by a virulent leaf rust race [11]. It is expected that durable adult plant partial resistance genes similar to *Lr34*, *Lr46*, and *Lr67* are present in the oat gene pool.

The oat line MN841801 has consistently demonstrated resistance to various *P. coronata* populations in rust nurseries for more than 35 years [12]. Chong [13] in a preliminary study of the partial resistance of MN841801 concluded that this line carries two APR genes with additive effects. A number of QTL controlling APR [5,14-17] have been detected in oat during the last two decades. Portyanko et al. [5] found four major QTL and three minor QTL for APR contributed by MN841801-1 in a RIL population derived from the cross MN841801-1 × Noble-2. A recent study by Acevedo et al. [17] validated these APR QTL and discovered one new QTL from the same cross. In total, eight QTL associated with MN841801-1 alleles were detected in previous studies. These studies used amplified fragment length polymorphism (AFLP), restriction fragment length polymorphism (RFLP), and diversity array technology (DArT) markers for mapping the QTL.

A unique feature of single nucleotide polymorphism (SNP) markers is the modest cost per data point and speed of data acquisition [18]. One versatile SNP detection system is the Illumina Infinium assay (Illumina Inc., San Diego, CA). The high-throughput nature of the Illumina assay makes it a good platform for genotyping bi-parentally derived populations used in QTL mapping [19]. Collaborative research in the oat research community has led to the identification of numerous oat SNPs and the development of a 6 K oat Infinium SNP beadchip array [20].

The objectives of this research were to: 1) develop a whole-genome genetic linkage map for an AC Assiniboia/MN841801 population using the 6 K oat and 90 K wheat Infinium SNP genotyping assays, 2) detect and characterize QTL for crown rust APR from MN841801 in the AC Assiniboia/MN841801 population, 3) validate the QTL using KASP SNP genotyping in two additional RIL populations (AC Medallion/MN841801 and Makuru/MN841801), and 4) predict orthologous wheat APR genes.

## Methods

### Plant materials

The MN841801 experimental oat line was developed by Paul Rothman at the University of Minnesota in the early 1970s, and has demonstrated to be resistant to diverse populations of crown rust in rust nurseries for over 35 years [12]. MN841801 has a complex pedigree (MN841801 Florad/Coker 58-7/3/CI7558//Black Mesdag/Aberdeen 101). Seed used in the present study was provided by Kurt Leonard (Cereal Pathology Laboratory, St. Paul, MN) in 1995. AC Assiniboia and AC Medallion are Canadian spring milling oat varieties with the crown rust resistance genes *Pc38*, *Pc39*, and *Pc68* [21,22]. Makuru is an oat variety from New Zealand that is susceptible to all known Canadian races of *P. coronata*. Three RIL populations were used in the study: AC Assiniboia/MN841801 (AsbMN) consisting of 163 F_8_-derived RILs, AC Medallion/MN841801 (MedMN) consisting of 156 F_6_-derived RILs, and Makuru/MN841801 (MakMN) consisting of 160 F_7_-derived RILs. All crosses were made in growth chambers at the Cereal Research Centre, Agriculture and Agri-Food Canada, Winnipeg, MB. The RIL populations were developed by single seed descent and raised in growth chambers. All panicles in all generations were bagged to prevent out-crossing.

### Crown rust assessment

The AsbMN population was tested at the Cereal Research Centre in Winnipeg in 2001 (MB01), 2002 (MB02), and 2013 (MB13), and at the University of Saskatchewan in Saskatoon in 2011 (SK11), 2012 (SK12), and 2013 (SK13). The MedMN population was tested in Winnipeg in 2001 (MB01). The MakMN population was tested in Winnipeg in 2001 (MB01) and 2002 (MB02). For all populations, three-replicate lattice designs were employed in all environments. Each population was tested as a separate experiment in environments where multiple populations were evaluated. In Winnipeg, the plots were sown as 1 m rows with a row spacing of 0.34 m. In Saskatoon, plots were sown as hills with a row spacing of 0.3 m and spaced 0.6 m apart along the seed row. In Saskatoon, a spreader row of AC Morgan was sown every sixteen rows to increase inoculum in the nursery. In Winnipeg, a spreader row of Makuru, Victory, and/or AC Morgan was planted every sixth row.

Field nurseries were inoculated with *P. coronata* isolate CR251, identified to be race BRBB [13]. CR251 is virulent on all of the seedling resistance genes present in the parents used in the study [13]. MN841801 has partial resistance to CR251 at the adult plant stage [12,13]. Two to three inoculations were done within 10 days when plants in the spreader rows reached the fourth leaf stage. At each inoculation, 0.3 g of crown rust urediniospores were mixed with 300 ml light mineral oil (Bayol®, Esso Canada, Toronto, ON.) and sprayed onto spreader rows with a Herbiflex hand-held sprayer (Micron Sprayers Ltd., Bromyard, UK). In Winnipeg, a custom built, automated misting system was used to provide a fine mist at intervals throughout the growing season to promote germination of the urediniospores and spread of the rust across the entire experimental block. In Saskatoon, water was sprayed onto the spreader rows after the Bayol had evaporated and covered with white plastic sheets. This was to simulate dew formation and ensured germination of the urediniospores and infection of the spreader rows.

Crown rust disease assessments were made on flag leaves when the epidemic was estimated to have reached its peak based upon past experience. Disease severity (DS) was estimated using the modified Cobb scale [23]. DS is a measure of percentage of the leaf covered by infection (flecks and/or pustules). Pustules were classified into reaction class (RC): resistant (R, flecks), moderately resistant (MR, tiny pustules), moderately susceptible (MS, moderate sized pustules), and susceptible (S, large pustules). Combinations of RCs were also possible (eg. RMR, MRMS, MSS), where observed symptoms were intermediate between reaction classes. All RCs were converted into a numerical value: R = 0, RMR = 0.1667, MR = 0.3333, MRMS = 0.5, MS = 0.6667, MSS = 0.8333, and S = 1. RC measurements were not recorded in the MB13 environment. Coefficient of infection (CI) of each plot was calculated with the formula: CI = (DS × RC) / 100. Heading date was recorded in the SK13 environment.

Statistical analyses were performed with JMP Genomics 6.0 (SAS Institute Inc., Cary, North Carolina, USA). The Fit Model Platform was used for ANOVA and to calculate least-squares means. Oat line was considered a fixed effect, while environment, rep, and incomplete block were random effects. Least-squares means were used for correlation analysis of traits using the Multivariate Platform.

### Genotyping

Genomic DNA was prepared from freeze-dried leaf tissue using the DNeasy Plant DNA extraction kit (Qiagen, Toronto, Canada). DNA was quantified with PicoGreen stain (Molecular Probes, Inc., Eugene, Oregon, USA).

The AsbMN population and parents were genotyped with the 6 K oat [20] and 90 K wheat [24] Illumina Infinium SNP arrays (Illumina, San Diego, CA). The raw data were analyzed with GenomeStudio V2011.1 software (Illumina, San Diego, CA). The genotype calls were converted into allele scores for linkage mapping in Excel. Markers with greater than 10% missing data or strong segregation distortion were excluded from mapping.

Nine 6 K oat Infinium SNPs and five genotyping-by-sequencing (GBS) SNPs [25] surrounding the *QPc.crc-14D*, were converted to Kompetitive Allele Specific PCR (KASP) SNP genotyping technology (LGC Genomics LLC, Beverly, MA, USA). For each KASP SNP, two allele-specific forward primers (A1 and A2) and two common reverse primers (C1 and C2) were designed (Additional file 1: Table S1). Only one common reverse primer is used in the KASP assay. The second common reverse is available if the assay fails with the first common reverse primer. The newly developed KASP primers were used in validating *QPc.crc-14D* in two populations, MedMN and MakMN respectively. A panel of 43 North American oat lines was also used to test the utility of the newly developed markers in a broader set of germplasm. Details on these oat lines are presented in Additional file 1: Table S2. KASP genotyping assays were performed as described by Gnanesh et al. [6].

### Linkage, QTL, and comparative mapping

The linkage map was developed with the software MapDisto v. 1.7.7 [26]. Linkage groups were identified with a minimum LOD score of 4 and a maximum recombination fraction between markers of 0.2. Marker order was determined with the AutoMap function and different combinations of the Branch and Bound II and Seriation II ordering algorithms with Sum of Adjacent Recombination Fractions and Count criteria for ordering and rippling. The Kosambi mapping function [27] was used to calculate map distances (cM) from recombination fractions.

QTL mapping was performed on least-squares mean data of DS, RC, CI from each field disease nursery environment. Simple interval mapping based on maximum-likelihood was conducted using QGene v. 4.3.10 [28]. A permutation test with 1,000 iterations was conducted to determine a significance threshold for each trait. Single marker analysis was used to determine association (P < 0.05) between unlinked markers and traits. Linkage maps and QTL scans were drawn using MapChart v. 2.1 [29]. Additive effect was calculated as (female parent allele RIL mean – male parent allele RIL mean) / 2.

DNA sequences from the oat and wheat SNPs linked to *QPc.crc-14D* were compared to contigs in the wheat chromosome arm-specific survey sequence with BLASTN with an E-value threshold of e-10. The wheat survey sequence data were generated by the International Wheat Genome Sequencing Consortium (IWGSC, www.wheatgenome.org) and downloaded from Unité de Recherche Génomique Info website (URGI, http://wheat-urgi.versailles.inra.fr/). The oat SNP sequences were reported in Tinker et al. [20] and Wang et al. [24].

## Results

### Phenotypic analysis of parents and populations

**Figure 1:**
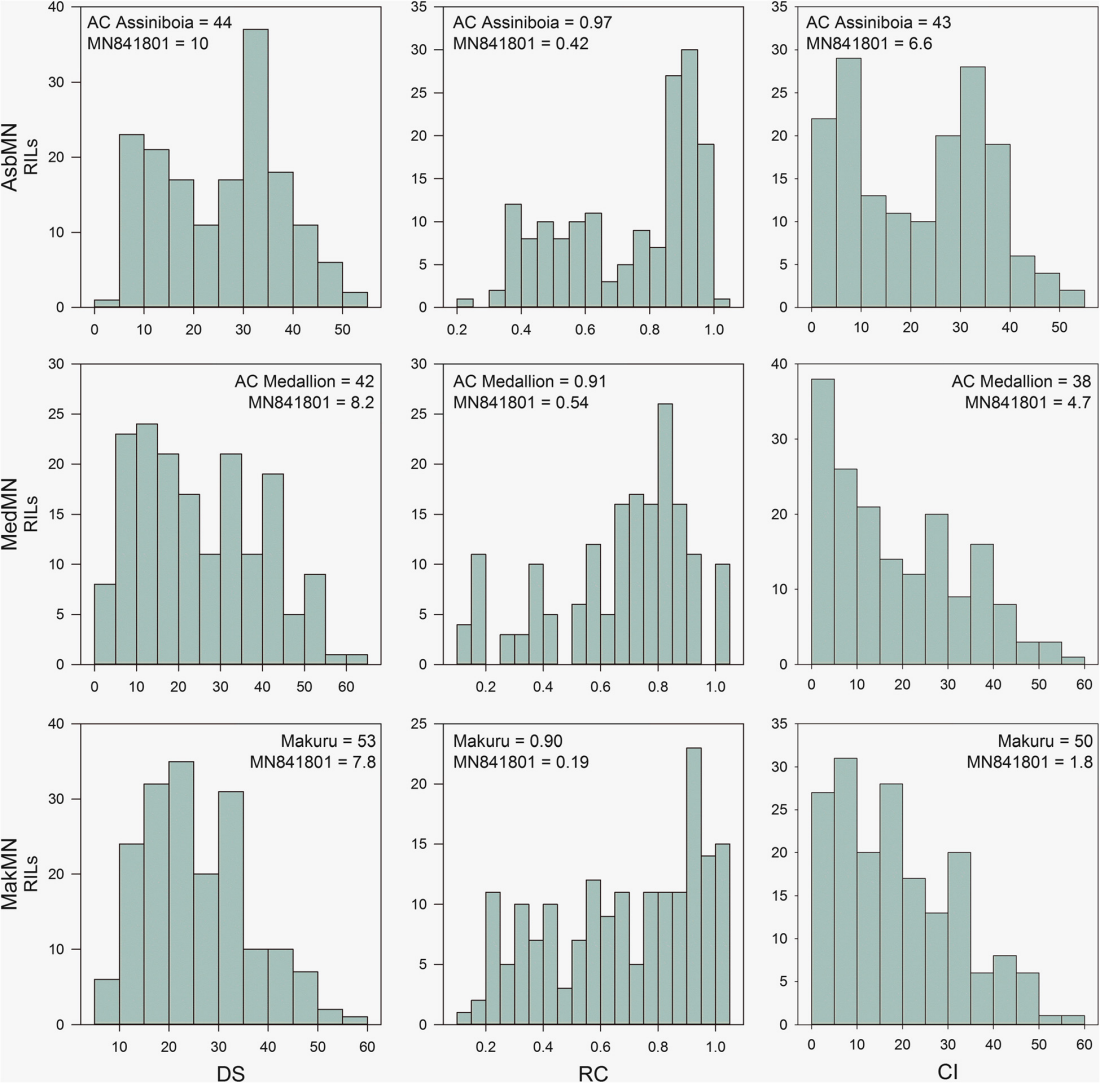
**Histograms of mean flag leaf crown rust disease severity (DS), reaction class (RC), and coefficient of infection (CI) for the RIL populations AC Assiniboia/MN841801 (AsbMN), AC Medallion/MN841801 (MedMN), and Makuru/MN841801 (MakMN) pooled over six, one, and two environments, respectively.** Means of the parents are indicated.

Distributions of mean flag leaf crown rust DS, RC, and CI for each of the RIL populations are reported in Figure 1. Table 1 provides additional data on flag leaf crown rust DS for each population in each environment. MN841801 was among the most resistant genotypes of each RIL population. AC Assiniboia, AC Medallion, and Makuru were among the most susceptible genotypes of each respective RIL population in terms of crown rust RC. Makuru was among the most susceptible genotypes of the MakMN population with regard to DS and CI, whereas AC Assiniboia and AC Medallion were somewhat less susceptible in terms of crown rust DS and CI. This suggested transgressive segregation for crown rust resistance in the AsbMN and MedMN populations. Heading date data for the AsbMN in Saskatoon 2013 was approximately normally distributed (data not shown). AC Assiniboia (Julian date = 202.0) and MN841801 (Julian date = 199.7) had similar heading dates, approximately the mean of the population. Heading date in the AsbMN population ranged from 197.5 to 208.2 on the Julian calendar.

**Table 1:** **Least-squares means of parents and descriptive statistics of the RIL populations for flag leaf crown rust disease severity (DS) in field crown rust nurseries**

**Environment** ^**a**^	**Minimum**	**Mean**	**Maximum**	**Parent means**	
AsbMN RIL population				AC Assiniboia	MN841801
MB01	1.5	12.7	40.4	25.6	4.4
MB02	2	16.7	38.6	33.5	3.4
SK11	2.8	34.8	78.4	54.5	9.3
SK12	5.5	25.3	78.6	39	7.8
MB13	1.4	42.4	91.4	75.3	26.8
SK13	−3.2	20.9	68.5	38.7	8.8
Mean	3.5	25.5	51.6	44.3	10.2
MedMN RIL population				AC Medallion	MN841801
MB01	0.7	23.8	63.3	42.2	8.2
MakMN RIL population				Makuru	MN841801
MB01	5.4	31.3	70	71	9.3
MB02	5	19.7	46.7	35	6.4
Mean	5.4	25.5	56.7	53	7.8

^a^SK = Saskatoon, SK; MB = Winnipeg, MB; 01 = 2001, 02 = 2002; 11 = 2011; 12 = 2012; 13 = 2013; mean = pooled mean for the trait over all environments.

### Linkage mapping

A total of 1,684 SNP markers were mapped on the AsbMN RIL population using Illumina Infinium 6 K oat (1,207 SNPs) and 90 K wheat (477 SNPs) SNP arrays. Chi-square (*χ*^2^) analysis was performed on the genotypic data to test the null hypothesis for the expected 1:1 Mendelian segregation on all of the scored markers. Of these, 15 (0.8%) markers were significantly skewed at the 0.01 level (four wheat SNPs and 11 oat SNPs). In total, 1,684 SNPs were mapped to 45 linkage groups belonging to 21 oat chromosomes. Only four SNPs were unlinked. The LGs were assigned to chromosomes based on the oat consensus map [30]. The basic information pertaining to the linkage groups (LGs) is presented in Additional file 1: Table S3. The AsbMN linkage map was 1,540 cM in length with a mean of 1.1 markers per cM. The complete linkage map is presented in Additional file 1: Table S4. Chromosome 5C had the most markers (180 markers). The wheat Infinium SNPs scored accurately and integrated in the linkage map without causing any disruptions or expanding the map. The wheat SNPs were interspersed throughout the genetic map as expected.

### Identification of APR QTL

**Figure 2:**
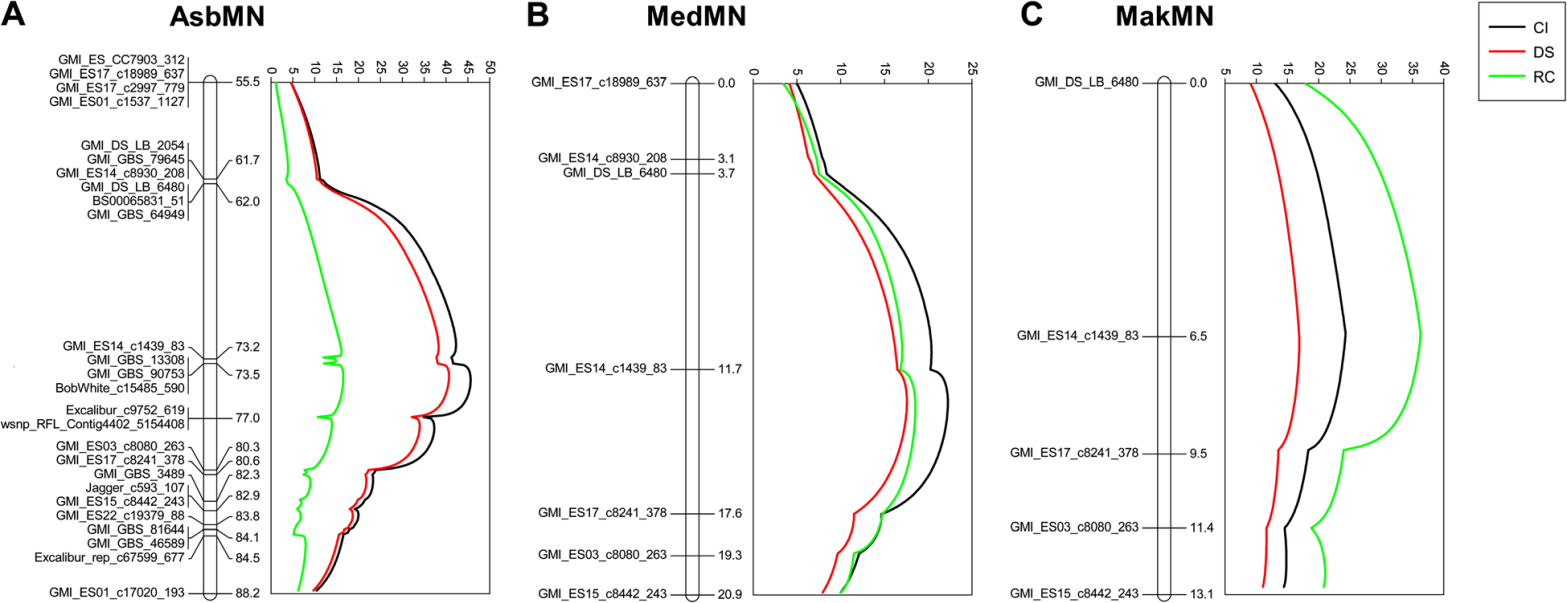
**Simple interval mapping (SIM) QTL scans revealing** ***QPc.crc-14D*** **on oat chromosome 14D.** QTL analysis of APR is based upon data averaged environments for disease severity (DS), reaction class (RC), and coefficient of incidence (CI) in the mapping populations: **A** AC Assiniboia/MN841801 (AsbMN), **B** AC Medallion/MN841801 (MedMN), and **C** Makuru/MN841801 (MakMN).

**Figure 3:**
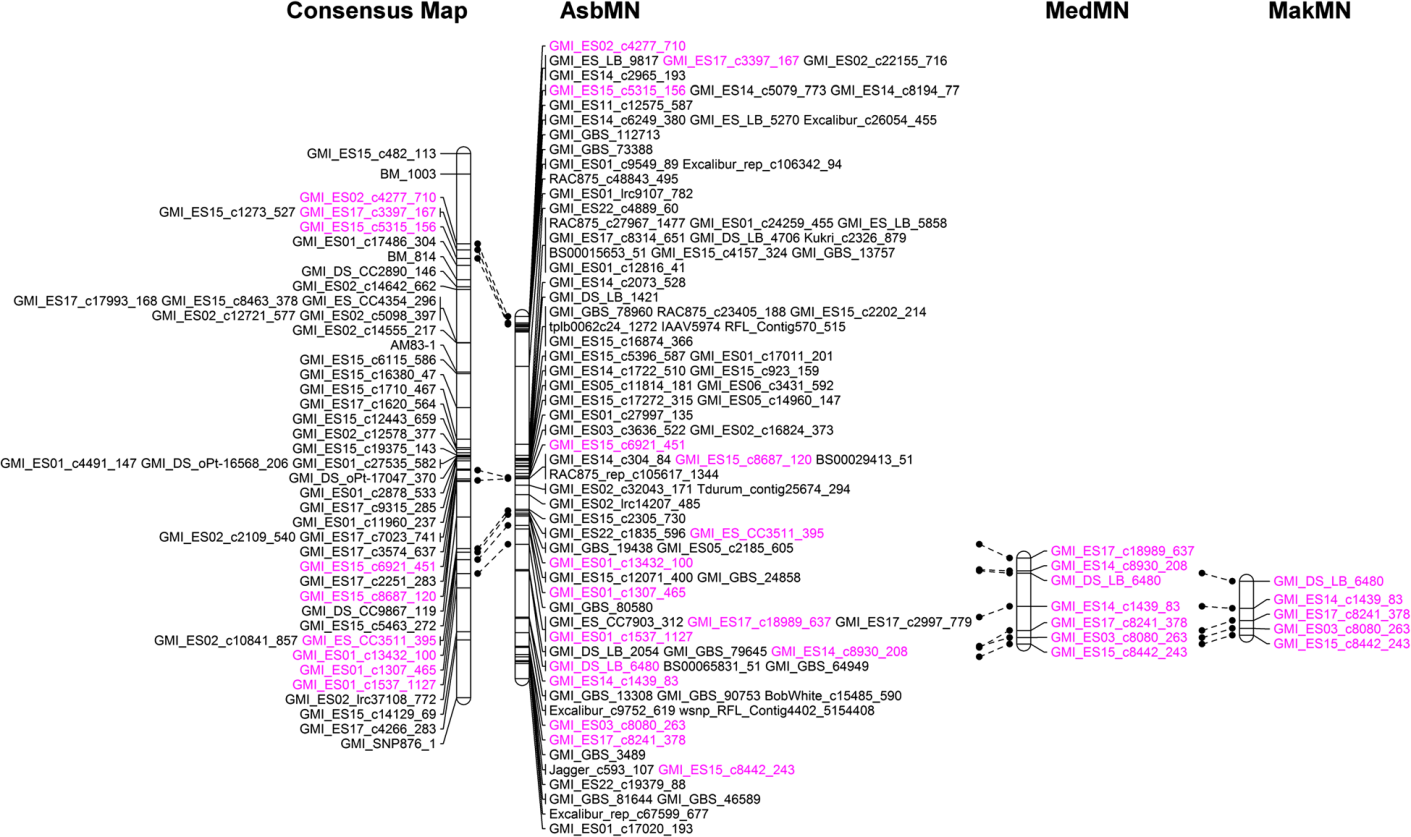
**Comparison of chromosome 14D linkage maps from the AC Assiniboia/MN841801 (AsbMN), AC Medallion/MN841801 (MedMN), and Makuru/MN841801 (MakMN) mapping populations with the oat consensus map [** 30 **].** The positions of common markers between two different LGs are bold and italicized.

Simple interval mapping identified a major QTL for crown rust APR (DS, RC, CI) in the AsbMN population in all six environments evaluated (Table 2). This QTL is named *QPc.crc-14D* and is present on oat chromosome 14D. The highest LOD score and R^2^ value for *QPc.crc-14D* were 49.6 and 75.8%, respectively, for RC in environment SK11 (Table 2). *QPc.crc-14D* is well marked with multiple SNPs underlying the genetic region. QTL scans showing the occurrence of *QPc.crc-14D* based upon simple interval mapping for DS, RC, and CI data averaged over environments is presented in Figure 2a. Although there were differences in the phenotypic variation explained by *QPc.crc-14D*, the results clearly suggest that nearly all the detectable variation for APR in the AC Assiniboia/MN841801 population is due to the presence of one major QTL. The major QTL *QPc.crc-14D* was also significant when the dataset was analyzed by multiple interval mapping. The multiple interval mapping analyses are not presented because the results were the same as simple interval mapping. Simple interval mapping and multiple interval mapping did not identify any other significant QTL for crown rust resistance in the AsbMN population. Comparison of chromosome 14D of the AsbMN map revealed 9 common markers with chromosome 14D of the oat consensus map [30]. The order of these common markers was congruent between the genetic maps (Figure 3).

**Table 2:** **Effect of the major APR crown rust resistance QTL** ***QPc.crc-14D*** **on coefficient of infection, disease severity, and reaction class in the AC Assiniboia/MN841801 (AsbMN) population**

	**Coefficient of Infection**						**Disease severity**							**Reaction class**					
**Statistic**	**Mean** ^**a**^	**SK11**	**SK12**	**SK13**	**MB01**	**MB02**	**Mean**	**SK11**	**SK12**	**SK13**	**MB01**	**MB02**	**MB13**	**Mean**	**SK11**	**SK12**	**SK13**	**MB01**	**MB02**
LOD	45.6	47.0	8.4	28.9	14.0	26.6	40.7	39.1	8.9	24.8	9.6	19.0	39.4	16.5	49.6	10.4	44.4	22.4	33.6
Additive effect	11.86	19.53	6.82	10.9	4.41	8.25	10.35	16.6	6.51	9.17	3.37	5.37	21.74	0.18	0.26	0.11	0.22	0.17	0.30
R^2^ (%)	72.9	73.9	21.4	56.2	32.9	53.2	68.8	67.3	22.4	50.8	24.0	41.9	68.1	37.7	75.8	25.7	71.9	47.3	61.8
Position^b^	74.5	74.7	73.1	75.5	73.1	71.0	74.3	74.4	73.1	75.7	73.1	74.1	74.7	74.4	74.6	70.4	75.1	73.9	70.0
LOD Threshold^c^	3.33	3.15	2.90	3.12	3.02	3.13	3.29	3.25	3.10	3.22	3.17	3.23	3.23	2.50	3.16	3.13	3.07	3.08	3.22

^a^Mean = pooled mean for the trait over all environments; SK = Saskatoon, SK; MB = Winnipeg, MB; 01 = 2001, 02 = 2002; 11 = 2011; 12 = 2012; 13 = 2013.

^b^Position in cM on the chromosome 14D linkage map.

^c^LOD significance threshold based upon 1,000 permutations (α = 0.05).

The additive effect of the QTL was 10.3 for DS in the pooled dataset, such that the mean of MN841801 class was 20.6 units lower than the AC Assiniboia class for DS using the modified Cobb scale. In addition, the additive effect of *QPc.crc-14D* on RC was 0.18 in the pooled mean dataset. Over the five environments, RILs with the MN841801 allele had an RC that was 0.36 lower than RILs with the AC Assiniboia allele. Overall, *QPc.crc-14D* affected crown rust severity and reaction class thereby affecting sporulation of the fungus by reducing the number of rust pustules and their size.

One QTL for heading date was identified in the AsbMN population on chromosome 13A (linkage group 16). However, it should be emphasized that heading date data was only collected in a single environment. The QTL peak was at the end of the linkage group at GMI_ES14_c7220_194, such that the QTL peak was not well defined. The LOD peak was 3.4 and explained 9.2% of the phenotypic variance. The LOD significance threshold was 2.9 for heading date in the SK13 environment. The additive effect was 0.74, such that RILs homozygous for the AC Assiniboia allele were 1.5 days later to heading than RILs homozygous for the MN841801 allele. No heading date QTL were detected on chromosome 14D.

### Validation of *QPc.crc-14D*

Fourteen SNPs surrounding *QPc.crc-14D* were evaluated for conversion to the KASP SNP assay. Seven and five SNPs were successfully scored and mapped on the two additional mapping populations MedMN and MakMN, respectively (Additional file 1: Tables S5 and S6). The SNP GMI_GBS_90753 was one of the nearest to the QTL peak, but could not be converted into a KASP assay because the available DNA sequence flanking the SNP was too short. Least-squares means of parents and descriptive statistics of AC Medallion/MN841801 (MedMN) and Makuru/MN841801 (MakMN) RIL populations for flag leaf crown rust DS are presented in Table 1. *QPc.crc-14D* mapped to the same region of chromosome 14D in the MedMN and MakMN populations as in the AsbMN population (Figures 2 and 3). GMI_ES14_c1439_83 was located at or near the QTL peak in the MedMN and MakMN populations in the environments tested. Again, *QPc.crc-14D* had a similar additive effect and explained similar amounts of phenotypic variation for flag leaf crown rust DS, RC, and CI in all of the populations and in all environments (Figure 2, Table 3).

**Table 3:** **Effect of the major crown rust APR QTL** ***QPc.crc-14D*** **on coefficient of infection, disease severity, and reaction class in the AC Medallion/MN841801 (MedMN) and Makuru/MN841801 (MakMN) RIL populations**

	**MedMN**			**MakMN**								
	**CI** ^**a**^	**DS**	**RC**	**CI**			**DS**			**RC**		
**Statistic**	**MB01** ^**b**^	**MB01**	**MB01**	**Mean**	**MB01**	**MB02**	**Mean**	**MB01**	**MB02**	**Mean**	**MB01**	**MB02**
LOD	22.5	17.6	18.5	24.3	18.0	26.7	16.9	11.7	18.7	36.3	26.7	37.4
Additive effect	10.39	9.76	0.166	9.58	11.19	8.00	6.90	8.10	5.78	21.3	20.9	0.23
R^2^ (%)	48.1	40.5	42.1	50.3	40.5	53.6	38.5	28.7	41.6	64.8	53.7	65.9
Position^c^	13.0	13.0	13.3	6.4	6.4	6.4	6.7	7.0	6.1	6.4	6.4	5.5

^a^ *CI* coefficient of infection, *DS* disease severity, *RC* reaction class.

^b^SK = Saskatoon, SK; MB = Winnipeg, MB; 01 = 2001, 02 = 2002; 11 = 2011; 12 = 2012; 13 = 2013; mean = pooled mean for the trait over environments.

^c^Position in cM on the chromosome 14D linkage map for the specific cross.

### Comparative mapping

Comparative analysis using SNPs linked to *QPc.crc-14D* identified synteny with group 2 chromosomes of wheat. A set of 11 oat and wheat SNPs, mapped on oat chromosome 14D in the region of *QPc.crc-14D*, showed highly significant BLASTN matches (E-value < E-10) with contigs in the wheat chromosome arm-specific survey sequence (Additional file 1: Table S7). The best BLASTN hits were all located on group 2 chromosomes of wheat. SNPs mapping to the 48.5 to 73.5 cM region of AsbMN chromosome 14D hit wheat contigs from the group 2 short arm libraries, while the SNPs mapping to 77.0 to 84.5 cM region of AsbMN chromosome 14D hit wheat contigs from the group 2 long arm libraries. This result indicated that *QPc.crc-14D* is syntenic with the centromeric regions of wheat group 2 chromosomes.

### Postulation of *QPc.crc-14D*

Five SNPs linked to *QPc.crc-14D* were tested on a panel of 43 oat lines. Three of these SNPs were not diagnostic based on the alleles identified in the highly susceptible oat varieties AC Morgan and Makuru. AC Morgan carried the MN841801 allele for SNPs GMI_ES03_c8080_263 and GMI_ES17_c8241_378 (Additional file 1: Table S2). Makuru possessed the MN841801 allele for GMI_ES14_c8930_208. The MN841801 haplotype for the two remaining SNPs was present in the oat lines 02P07-BC1A and W02203, both of which have MN841801 in their pedigrees and are likely carriers of *QPc.crc-14D*.

## Discussion

The present study identified a major QTL for adult plant crown rust resistance in the oat line MN841801. *QPc.crc-14D* was identified in three bi-parental mapping populations and was significant in every crown rust nursery in which these populations were evaluated for all measures of disease symptoms (DS, RC, and CI). The resistance observed in these field trials was based upon adult plant resistance because the *Puccinia coronata* isolate CR251 is virulent on MN841801 at the seedling stage [13]. The resistance conferred by *QPc.crc-14D* is best described as partial resistance since the RC of lines with the QTL is MR to MRMS in the field disease nurseries. In addition, the crown rust resistance in MN841801 has remained stable for more than 30 years [12]. *QPc.crc-14D* may function in a similar manner to known durable adult plant leaf rust resistance genes in wheat, such as *Lr34*, *Lr46*, and *Lr67* [8-10]. *Lr34* has been sequenced and is encoded by an ATP-binding cassette (ABC) transporter [31]. Comparison of this study with other studies of crown rust resistance in oat is very difficult because there are no markers in common between the studies (RFLPs vs. SNPs) and the linkage maps in previous studies were not anchored to chromosomes based upon cytogenetic evidence. *QPc.crc-14D* is assigned to chromosome 14D based upon comparison with an anchored genetic map [30]. *QPc.crc-14D* is the first major crown rust resistance QTL detected in the oat line MN841801 that has consistent effects across environments.

Previous research on the crown rust resistance in MN841801 suggested that the resistance was either qualitative [13] or highly quantitative [5,17]. Chong [13], in a preliminary study on the segregation of partial resistance in the same AsbMN RIL population, concluded that MN841801 carries two additive APR genes effective against isolate CR251. This conclusion was based on rust reactions observed on the RILs after a single inoculation in growth chamber experiments. Rust reactions resulting from a single cycle of infection are be useful for examining infection types but would not be useful for detecting resistance which expresses quantitatively over multiple disease cycles. This might explain the discrepancy in findings between the preliminary study (two additive APR genes) and the present study (single major QTL for APR). Alternatively, it is possible that a second undiscovered APR QTL exists in the AsbMN population, since the linkage map does not span the complete oat genome. Portyanko et al. [5] identified four major (*Prq1a*, *Prq1b*, *Prq2*, and *Prq7*) and three minor (*Prq3*, *Prq5*, and *Prq6*) APR QTL using a 230 marker linkage map derived from the population MN841801-1/Noble-2. The same population was evaluated in an additional seven field environments and two greenhouse tests [17]. The same seven QTL were detected plus one additional QTL (*Prq8*), and again, QTL were not consistently detected across environments. All QTL, except *Prq1b*, were detected in one or more field environment inoculated with an isolate virulent on both parent in seedling tests [17]. However, *Prq1b* was detected in adult plant tests inoculated with an isolate virulent on both parent in seedling tests [5,17]. Major QTL for flowering time overlapped with *Prq1a*, *Praq1b*, and *Prq7* [5,17], which could indicate a pleiotropic effect on flag emergence date and crown rust symptom development. These resistance QTL were still detected in the adult plant greenhouse tests in Portyanko et al. [5], but not in Acevedo et al. [17]. The adult plant greenhouse tests should have eliminated effects of flag leaf emergence date on crown rust symptoms in both studies. *Prq2*, *Prq5*, and *Prq8* were the most consistently detected QTL in the field and greenhouse tests inoculated with isolates virulent on the parents of the population in seedling tests [5,17]. However, none of the major QTL described in Portyanko et al. [5] and Acevedo et al. [17] had the same consistent dramatic effect as *QPc.crc-14D*. The crown rust data reported in Portyanko et al. [5] was based upon inoculation with a mixture of crown rust races, which could have confounded seedling and adult plant resistance since three seedling genes were postulated in MN841801 when inoculated with CR250 [13]. Some of the field crown rust nurseries in Acevedo et al. [17] were inoculated with isolates virulent on the parents at the seedling stage, which would overcome this problem. The discrepancies in these results are remain difficult to explain. Possibly the original MN841801 line was heterogeneous and the MN841801 plant used to generate the MN841801-1/Noble-2 population was genetically different than the MN841801 lineage used to develop the AsbMN, MedMN, and MakMN populations.

SNP markers are attractive for use in genetic mapping and marker-assisted breeding because they can be scored in parallel assays at favorable costs. In this study, the 6 K oat and 90 K wheat Infinium SNP arrays were used to develop a linkage map for the AsbMN population. The AsbMN linkage map consisted of 1,684 loci and spanned 1540 cM, or 1.1 loci per cM. This compares favourably with the oat consensus map [30], which has 1,054 loci over 1,839 cM, or 0.6 loci per cM. The 90 K wheat Infinium SNP array was investigated to increase the coverage of the oat genome and to investigate synteny with the wheat genome. Markers derived from the wheat SNP array (477 loci) comprised 28% of the markers on the AsbMN linkage map, which was a significant source of additional markers. However, the wheat 90 K SNP array consists of 81,587 SNPs (attempted bead types) such that only 0.58% of the attempted bead types resulted in a locus that could be mapped.

The 90 K wheat SNP array was useful for exploring the syntenic relationships between the oat and wheat genomes. Eleven wheat SNPs near *QPc.crc-14D* were mapped to a 36 cM region on oat chromosome 14D. The linear order of these SNPs on the AsbMN genetic map was consistent with the predicted chromosome arm placement of the SNPs in the chromosome arm-specific wheat survey sequence. The peak of *QPc.crc-14D* was located at the centromere of wheat group 2 chromosomes. The adult plant stripe rust resistance gene *Yr16* is located in the centromeric region of wheat chromosome 2D [32,33], which suggests that *QPc.crc-14D* is orthologous to *Yr16* in wheat. Fine mapping of these genes should determine whether or not this is the case. The development of DNA markers for *Yr16* should be beneficial for *QPc.crc-14D*, and vice-versa. However, sequencing of these genes may be hindered by their apparent proximity to the centromere of their respective chromosomes.

The SNPs GMI_DS_LB_6480 and GMI_ES14_c1439_83 have potential as diagnostic markers for *QPc.crc-14D*. The scores of these SNPs were different on the panel of oat lines tested. GMI_ES14_c1439_83 is located nearest to the QTL peak in all three populations suggesting that it will be the most broadly applicable for marker-assisted selection. However, other linked SNPs may be useful in specific crosses. The present data do not reveal which SNP is most diagnostic. Additional testing should be conducted on a large panel of crown rust susceptible oat lines. With these markers, deployment of *QPc.crc-14D* will be feasible by oat breeders. If the QTL confers truly non-race-specific resistance, then it could be deployed either singly or with other crown rust resistance genes. On its own, *QPc.crc-14D* provided considerable protection from *P. coronata* isolate CR251 in this study. Pyramiding *QPc.crc-14D* with other crown rust resistance genes should be an effective management method. As with all disease resistance genes, durability can only be proven after widespread deployment of the gene in commercial fields. Since *QPc.crc-14D* has not been widely deployed to our knowledge, the durability of the QTL is not known.

The MN841801 haplotype for the SNPs GMI_DS_LB_6480 and GMI_ES14_c1439_83 was only present in the oat lines 02P07-BC1A, W02203, and CIav 8361. MN841801 is in the pedigree of 02P07-BC1A and W02203. The pedigree of CIav 8361 is unknown, but this line was also developed by the University of Minnesota (as was MN841801). The CIav and PI lines present in the oat panel are believed to possess APR to crown rust based upon data from field and seedling tests (Drs. Michael Bonman, Marty Carson, and James Chong, unpublished data). Other than CIav 8361, none of these lines had the MN841801 haplotype for the two most predictive SNPs for *QPc.crc-14D*. This suggests that different crown rust APR genes are likely present in these lines and these lines should be investigated further.

## Conclusion

Crown rust resistance is a high priority for oat breeding research on a global basis. The development of durable crown rust resistance is a key target given the rapid breakdown of crown rust resistance based upon race-specific seedling resistance genes. A major partial resistance APR QTL, named *QPc.crc-14D*, was detected in the AsbMN population with the resistance allele contributed by MN841801. Numerous SNPs surrounding the QTL were converted to KASP SNP assays and successfully validated in the MedMN and MakMN populations. SNPs suitable for selection of *QPc.crc-14D* were identified. Comparative mapping with wheat suggests that *QPc.crc-14D* is orthologous to the stripe rust APR gene *Yr16*.

### Availability of supporting data

All supporting data are included as an additional file.

## Additional file

Additional file 1:
**This file contains seven supplementary tables.** **Table S1** outlines KASP primers. **Table S2** provides pedigree information and SNP haplotype data on oat lines in the postulation study. **Table S3** summarizes the AsbMN linkage map. **Tables S4, S5, and S6** present the linkage maps for the AsbMN, MedMN, and MakMN populations. **Table S7** presents BLAST results of oat and wheat SNPs versus the wheat chromosome arm-specific survey sequence.

## Abbreviations

ABC: ATP-binding cassette
AFLP: Amplified fragment length polymorphism
APR: Adult plant resistance
AsbMN: AC Assiniboia/MN841801
CI: Coefficient of infection
DArT: Diversity array technology
DS: Disease severity
GBS: Genotyping-by-sequencing
KASP: Kompetitive Allele Specific PCR
MakMN: Makuru/MN841801
MB01: MB02, MB13, disease nursery at Winnipeg, MB 2001, 2002, and 2013, respectively
MedMN: AC Medallion/MN841801
QTL: Quantitative trait locus
RC: Reaction class
RFLP: Restriction fragment length polymorphism
RIL: Recombinant inbred line
SNP: Single nucleotide polymorphism
SK11, SK12, SK13: Disease nursery at Saskatoon, SK 2011, 2012, and 2013, respectively
